# Mr. Simmons, on Arsenic in Cancer

**Published:** 1801-07

**Authors:** W. Simmons

**Affiliations:** Manchester


### *To the Editors of the Medical and Physical Journal.*

GENTLEMEN,

TO thofe of your readers who have taken an intereft in the event of the cafe of Saunders, a cancer-patient, then under a courfe of arfenic, the fequel of my narrative, contained in the xxvth Number of your Work, may not be unacceptable. At that time I had pretty clearly afcertained that arfenic did poffefs the the property of eafing the pain in the ulcerated parts; and to enable me to continue this falutary influence, fhould the arfenical folution difagree, I had it in contemplation to combine opium along with it, or digitalis, according to the nature of the complaint which might be excited by its continued exhibition. Soon after my former report, my patient complained of an uneáfinefs at her ftomach, and of a fevere head-ach, for which, under a fufpicion that they might be occafioned by the folution, I added an equal portion of laudanum, being unwilling to interrupt the ufe of the former, left the beneficial impreffion on the fyftem made by it fhould be loft. In this form however it difagreed with her fo much more than before, that I fufpended the ufe of it altogether, and had recourfe to remedies fuited to the feveral indications. Ten days had elapfed before I could again employ the arfenic, during which time the ulceration had confiderably extended, and latterly fhe had fuffered feverely from a moft diftreffing burning fenfation, that fpread over the whole ulcerated furface. The drops were now given in pepper-mint water, to which was afterwards added a little tincture of lavender, as a further corrective. They fat eafy on her ftomach thus combined, and were continued till within a few days of her death, which happened early in the morning of the 26th of May, in confequence of a diforder brought on by taking cold, and upwards of ten months from beginning the ufe of the arfenic.

Till the fufpenfion of the folution above ftated, the ulcerated parts were free from pain, and the fenfation of heat was removed in a few days after renewing it. Not fo, however, with the knots; when active, which fome of them generally were, the pain in them was extremely acute; one while fhe thought them more fo againft a change in the weather; at another they were worfe without any affignable caufe of aggravation. Neverthelefs, they had not multiplied very numeroufly, and thefe were chiefly around the ulcerated furface, only two or three having extended towards the right breaft acrofs the foffa of the bofom, and the reft had taken the direction of the axilla. Without any difcoverable breach in the folids, fhe occafionally loft a fmall quantity of blood from the knots, by which fhe thought the pain confiderably abated. Profiting by this hint I ventured to apply a leech or two, from time to time, when the pain was unufually fevere in any of them, and the bleeding never failed to afford her relief. I had fome fcruples relative to the propriety of employing leeches in this cafe, left their bites fhould degenerate into ulcers, which they are apt enough to do, and thereby an extenfion of the difeafe might be produced; till it occurred to me, that fhould ulceration enfue, the difeafe would would then be brought more immediately under the influence of the remedy, and a painful diſorder would be converted into one unattended with pain. But the bites healed without ulcerating.

Where ſo much time is occupied in conducting a caſe through its different ſtages, a variety of incidents muſt neceſſarily occur, not at all important to the main object in view; the recounting of which would only produce fatigue to the reader, and likewiſe increaſe the conſumption of paper. In the preſent inſtance, therefore, I have confined myſelf to a ſummary of the facts; the remainder of which I ſhall now proceed to narrate, together with the appearances on diſſection.

Though the burning ſenſation, ſo characteriſtic of the cancerous ulcer, was removed in a few days by the repetition of the arſenic, the ſpace loſt by the extenſion of the ulceration was never entirely regained. Little diſpoſition to heal appeared for ſeveral weeks after, and as the drops gave no diſturbance, the doſe was augmented to 14 three times a day; which however ſhe continued but for a few days in the increaſed quantity, becauſe, as the ſkinning proceſs was reſumed, it was thought more prudent to deſiſt, and ſhe again took it in the former doſes. I will not venture to determine abſolutely, whether the augmented doſes forwarded the cicatrization at this time, or whether it was the reſult of the preceding ones now acting more powerfully on the ſyſtem; but the healing went on until the ulcer was reduced to nearly its former dimenſions; and, at the time of her death, the ulcer was not larger than the palm of my hand.

There was no occaſion to employ the digitalis.

The ſame external application was continued all along after my laſt report.

In proſecuting the recital of facts, I muſt obſerve that ſhe ſuffered a good deal of pain in the axilla on the left ſide, and in the left arm, in which an enlargement took place; and, before any viſible increaſe elſewhere, a tumefaction appeared over the knuckles of the hand on the ſame ſide, that was ſynchronous with the diſeaſe in the breaſt; ſo that when a more than uſual irritation prevailed in the latter, it was ſo diſtinctly marked by the increaſe of the former, that I could pretty accurately tell the ſtate of the ulceration by looking at the back of the hand. This ſynchronous action in the hand and breaſt continued for ſeveral weeks before the general enlargement of the limb manifeſted itſelf; which it then did by ſwelling from below upwards, particularly in the direction of the abſorbents on the inſide of the fore-arm, the trunks of which were perceptibly enlarged to the touch of myſelf and others. But that permanent change of ſtructure had taken place, either in the hand or fore-arm, was clearly evinced by the entire ſubſidence of the ſwelling ſoon after death; and the whole muſt conſequently have been cauſed by an irritation originating at the other extremity of the limb.

Although ſhe had been ſo long ill, the body was not at all emaciated, nor had the diſeaſe extended further than is above deſcribed.

On inſpecting the cavities, the only morbid appearance deſerving of notice was a ſlight ſcirrhoſity of the liver, that had not materially impeded the ſecretion of bile. In other reſpects the thoracic and abdominal viſcera were ſound, and the meſenteric glands were free from any enlargement. I was rather ſurpriſed not to find the lungs ſomewhat diſeaſed, becauſe ſhe had been troubled at times with a difficulty of breathing, attended with a mucous expectoration, even before the cancerous breaſt was removed; and had beſides the livid appearance on her lips, and other marks in her countenance of an impeded circulation through the lungs. All theſe ſigns however were fallacious, for the lungs were found of a beautiful grey colour, and not even an adheſion between them and the pleura had taken place. The trachea itſelf was narrower than uſual, though not remarkably ſo.

I have tranſcribed the formula employed in this caſe from the Pharmacopœia Chirurgica, and have ſubjoined it, for the benefit of thoſe who may not have that book in their poſſeſſion.

SOLUTIO ARSENICI.

℞. Arſenici pulveris ſubtiliſſimi, Kali præparati ſing. gr. xvj. Aq. diſtillat. ℥iv.

Theſe are to be digeſted together in a ſand heat, till the arſenic is completely diſſolved.

---

### *Noli me tangere.*

The quaint title of *Noli me tangere* has been uſed to deſignate a cancerous affection of the face, and is ſufficiently indicative of at leaſt a virulent diſeaſe. I know not whether I am warranted in applying it in the preſent inſtance, becauſe the event has proved favourable. But as the diſorder was a formidable one, and in the next ſtage of it might have been very truly denominated *let me alone*, I ſhall riſk the propriety of applying it, and proceed to detail the caſe.

Mary Lord was paſt the middle age, and mother of a large family. In December laſt ſhe inadvertently extracted a tooth, by entangling it in ſome twiſt ſhe was winding in the courſe of her occupation. A ſhort time after the accident, a tumour formed on the gum over the ſocket, and aſcended rapidly upwards. She felt no pain from it, nor was it even to the laſt at all painful to her. She applied at the Infirmary as an out-patient, a few months from the commencement of it, when it occupied nearly the whole of the left cheek. Beſides the tumour itſelf, there was ſo much contiguous ſwelling as to obſcure the real boundary, to aſcertain which, diſcutient applications were uſed, and with ſucceſs. The tumour then preſented a hard incompreſſible body, occupying the left ſide of the face, from the os malæ to the ſeptum naſi on the ſame ſide, and from its origin on the gum up to the inner canthus of the eye; and was likewiſe ſo firmly adherent to the maxillare ſuperius, and the adjoining bones, as, in the opinion of ſome, to be fixed immoveably. Under theſe circumſtances, a difference of opinion might reaſonably be entertained relative to the propriety and even practicability of removing it by an operation; and it was only after a ſecond conſultation that I was authorized to attempt it. In my own opinion, the tumour was ſeparable from the ſubjacent bones; and as it was free from pain, the ſkin free from diſeaſe, and no vital part was likely to be involved in the operation, I felt fully warranted in proceeding. Objections had been ſtarted on the ſuppoſition that the bones underneath might be carious; but, as in this caſe, the caries muſt have proceeded from preſſure, the removal of the cauſe might be ſuppoſed competent to the removal of the effect, and it would therefore be followed only by ſuperficial exfoliations. Thus fortified, I entered on the extirpation, which was accompliſhed without much difficulty, by making a longitudinal inciſion the whole length of the tumour, and diſſecting it out from the ſubjacent parts. The adheſions to the os malæ and contiguous bones were very ſtrong, but the perioſteum was not denuded from them; and though the whole was completely removed, no veſſel of importance or even requiring a ligature, was wounded. The divided parts united by the firſt intention, and ſhe was diſcharged cured in leſs than three weeks from the time of the operation.

Every conſiderable operation in ſurgery involves a queſtion of morality; and, to juſtify it, there ſhould not only be a probability of benefit to the patient, but alſo a reaſonable expectation that the operation itſelf may not prove mortal. To enter on an operation, which, from the nature of it, muſt be expected to end fatally, is deſignedly to put the patient to death, which, in principle, can never be warrantable; for, certainly, the *anceps remedium* has no reference to phyſical poſſibility. I have always conceived, that profeſſional evidence

 reſted refted on the calculation of probabilities, and therefore, whenever the chances were againft the fafety of an operation, that it ought to be abandoned. I am well aware that this doctrine might fometimes confign a patient to his fate, when a defperate effort might have faved him; but I am alfo confident, that fuch an occafional lofs would be a leffer evil to fociety than the contrary one; and, in my opinion, it is vindicable upon every principle of reafon, humanity, and found policy; and can never be fubverted by any argument founded on a calculation of diftant advantage.

No ulceration having here taken place, the whole of the difeafe was in all likelihood removed. Like cancer in other parts of the body, had there been abforption from it, the attempt at excifion would only have added another irritation to the one already exifting. Perhaps the too long delay before an attempt to remove cancerous tumours in the face, had deterred practitioners from encountering them, as, in that cafe, all means of relief muft have proved ultimately unfuccefsful. That the tumour in this cafe would foon have turned cancerous, there feems little doubt; when divided it was very hard in every part, and in fome almoft cartilaginous, intermixed with fpots of difcolouration.

I have the honour to be, &c.

